# Pharmacokinetics of single‐dose rivaroxaban under fed state in obese vs. non‐obese subjects: An open‐label controlled clinical trial (RIVOBESE‐PK)

**DOI:** 10.1111/cts.13853

**Published:** 2024-06-07

**Authors:** Majdoleen Alalawneh, Ahmed Awaisu, Ibtihal Abdallah, Hazem Elewa, Mohammed Danjuma, Kamal M. Matar, Akram M. ElKashlan, Yasser Elshayep, Fathy Ibrahim, Ousama Rachid

**Affiliations:** ^1^ College of Pharmacy, Health Sector Qatar University Doha Qatar; ^2^ Internal Medicine, Hamad General Hospital Hamad Medical Corporation Doha Qatar; ^3^ College of Medicine, Health Sector Qatar University Doha Qatar; ^4^ Department of Pharmacology & Therapeutics, Faculty of Pharmacy Kuwait University Kuwait City Kuwait; ^5^ Department of Biochemistry, Faculty of Pharmacy University of Sadat City Sadat City Egypt; ^6^ International Center for Bioavailability, Pharmaceutical, and Clinical Research Cairo Egypt; ^7^ Faculty of Pharmacy Al‐Azhar University Cairo Egypt

## Abstract

The evidence of rivaroxaban's pharmacokinetics in obese compared with non‐obese populations remains inconclusive. We aimed to compare the pharmacokinetic profile of rivaroxaban between obese and non‐obese populations under fed state. Participants who met the study's eligibility criteria were assigned into one of two groups: obese (body mass index ≥35 kg/m^2^) or non‐obese (body mass index 18.5–24.9 kg/m^2^). A single dose of rivaroxaban 20 mg was orally administered to each participant. Nine blood samples over 48 h, and multiple urine samples over 18 h were collected and analyzed for rivaroxaban concentration using ultra‐performance liquid chromatography coupled with tandem mass detector. Pharmacokinetic parameters were determined using WinNonlin software. Thirty‐six participants were recruited into the study. No significant changes were observed between obese and non‐obese participants in peak plasma concentration, time to reach peak plasma concentration, area under the plasma concentration–time curve over 48 h or to infinity, elimination rate constant, half‐life, apparent volume of distribution, apparent clearance, and fraction of drug excreted unchanged in urine over 18 h. Rivaroxaban's exposure was similar between the obese and non‐obese subjects, and there were no significant differences in other pharmacokinetic parameters between the two groups. These results suggest that dose adjustment for rivaroxaban is probably unwarranted in the obese population.

**Study Highlights**

**WHAT IS THE CURRENT KNOWLEDGE ON THE TOPIC?**

Despite the fact that obesity is a risk factor for the onset and recurrence of venous thromboembolism (VTE), and even though a large proportion of obese patients have VTE and thus need anticoagulation therapy, studies which have investigated rivaroxaban pharmacokinetics (PK) in obese subjects are limited. The lack of definitive evidence regarding rivaroxaban PK among the obese population is clearly reflected in clinical guidelines.

**WHAT QUESTION DID THIS STUDY ADDRESS?**

We investigated the PK and the coagulation profiles of rivaroxaban in obese vs. non‐obese subjects.

**WHAT DOES THIS STUDY ADD TO OUR KNOWLEDGE?**

In this prospective controlled clinical trial, PK and coagulation profiles after oral rivaroxaban 20 mg were mostly similar in obese subjects compared with non‐obese subjects.

**HOW MIGHT THIS CHANGE CLINICAL PHARMACOLOGY OR TRANSLATIONAL SCIENCE?**

This clinical PK study contributes to addressing the controversy in clinical practice that benefits the obese population by ensuring optimal pharmacotherapy with rivaroxaban.

## INTRODUCTION

Venous thromboembolism (VTE) is among the leading causes of cardiovascular death, after stroke and myocardial infarction.^1^, ^2^ The reported global incidence of VTE is around 10 million cases per year.^3^ On the other hand, atrial fibrillation (AF) is the most common sustained heart arrhythmia. According to a recent report by the European Society of Cardiology, AF affects an estimated 100 million people worldwide, resulting in a significant increase from previous estimates.^4^ The risk of recurrence of these incidences is also high, especially in the absence of secondary preventive therapies.^2^, ^5^ The overall risk of VTE or AF recurrence is approximately 30% over 10 years and 70% over 5 years, respectively.^6^, ^7^ Studies suggest that the prevalence of VTE and AF is increasing, partly due to an aging population and an increase in certain risk factors, such as obesity, hypertension, diabetes, and cancer.^8^, ^9^ In 2016, an estimated 650 million adults worldwide were obese, representing ~ 13% of the global adult population.^10^

In many cases, anticoagulation therapy is the gold standard approach for VTE treatment and prevention,^6^ and for AF treatment and stroke prevention.^11^ Due to their evident efficacy and safety, heparin, low molecular weight heparins (LMWHs), and warfarin have been the mainstay of anticoagulation therapy for many decades.^12^ However, these agents are associated with several adverse events and other limitations such as unpredictable pharmacokinetics (PK) and pharmacodynamics (PD), interactions with other drugs and food, and the need for frequent monitoring.^13^ Direct oral anticoagulants (DOACs) such as rivaroxaban and apixaban are a relatively new class of anticoagulants, which overcome the limitations of conventional anticoagulants.^14^ The recommendations of most clinical practice guidelines have identified DOACs as the first‐line therapy for the treatment/prevention of VTE, and as a stroke prophylaxis in patients with AF, when no contraindications for their use are present.^6^, ^15^

Rivaroxaban which acts via direct, selective, and reversible inhibition of factor Xa, thereby inhibiting the formation of fibrin clots and platelet activation, is among the FDA‐approved DOACs. Rivaroxaban is BCS class 2, which encompasses low solubility and high permeability drugs.^16^ It is practically insoluble in water/aqueous solutions (10 mg/L at 25°C).^17^ It has a molecular weight of 435.89 g/mol, a topological polar surface area of 116 Å^2^, six hydrogen bond acceptor and one hydrogen bond donor, which collectively contribute to rivaroxaban favorable membrane permeability and thus its good oral bioavailability of 80–100% with 2.5 and 10 mg doses.^18^, ^19^ The oral bioavailability decreases with higher doses of 20 mg, which can be enhanced under fed conditions.^20^ Rivaroxaban has a predictable pharmacokinetic profile.^21^ It is absorbed rapidly to achieve its maximum plasma concentration in 2–4 h (h), and has a half‐life range of 5–9 h in adults and 11–13 h in the elderly.^19^, ^21^ The drug binds reversibly and extensively (up to 95%) to plasma albumin with low‐to‐medium affinity to peripheral tissues, which explains the steady‐state volume of distribution value of 50 L (0.62 L/kg).^20^ It is primarily eliminated through renal excretion and hepatic metabolism. In urine, about 36% of the rivaroxaban total administered dose is recovered unchanged mainly via active secretion by P‐glycoprotein (P‐gp) and breast cancer resistance protein (ABCG2), and to a lesser extent by glomerular filtration, in a 5:1 ratio.^20^, ^22^ The remaining two‐thirds of the given dose is metabolized in the liver; either via oxidative biotransformation by cytochrome P450 enzymes, particularly CYP3A4 and CYP2J2 or by non‐CYP‐mediated amide bonds hydrolysis.^19^, ^20^ Half of the metabolites are excreted renally and half by the hepatobiliary route; no activity was associated with the circulating metabolites.^19^, ^20^ Rivaroxaban has a systemic clearance of approximately 10 L/hr, following intravenous administration.^20^

Generally, the PK parameters of a drug in normal‐weight individuals would be different from those of obese (BMI ≥30 kg/m^2^), particularly morbidly obese class III (BMI ≥40 kg/m^2^) individuals, and thus caution must be exercised as dose adjustment might be warranted in such population.^23^ Regarding rivaroxaban, despite the aforementioned PK properties in normal‐weight population, several previous studies have attempted to provide guidance on the PK changes of rivaroxaban in obese, including morbidly obese, subjects.^24^, ^25^, ^26^ Like other DOACs, dosing of rivaroxaban in morbidly obese patients is particularly an area of therapeutic dilemma and further research investigations are warranted.^27^, ^28^ This remains a challenge due to limited clinical and PK data to guide evidence regarding rivaroxaban dosing in a morbidly obese population.^26^, ^29^ While not necessarily the case for rivaroxaban, the scarcity of data on morbidly obese subjects can be partly explained by the fact that obese patients are usually excluded from most clinical trials, as some of the PK parameters in obese and morbidly obese patients such as protein binding, volume of distribution, and clearance might be different from those of non‐obese patients.^30^

Many attempts have been made to investigate the pharmacokinetics of rivaroxaban in the obese and morbidly obese populations.^24^, ^25^, ^26^ In their randomized controlled PK study, Kubitza et al. revealed that rivaroxaban PK parameters were comparable between morbidly obese and non‐obese populations, and thus no weight‐based dose adjustments were recommended for extreme weight populations (>120 kg & ≤50 kg).^31^ Similarly, Barsam et al. suggested that body weight has only little impact on rivaroxaban PK profile.^32^ On the other hand, Mueck et al. examined the PK profile of rivaroxaban in patients undergoing hip and knee replacement surgeries, and demonstrated that body weight has affected the volume of distribution of rivaroxaban.^33^ Furthermore, in a retrospective study, 28% of the morbidly obese patients had their rivaroxaban peak concentration below the 5th percentile of the peak concentration.^34^ However, the clinical relevance of a peak concentration below the 5th percentile of the peak concentration distribution is unknown, especially that the trough concentrations were not influenced in this study.^34^ Several other recent studies aimed to determine rivaroxaban's PK parameters in obese (including morbidly obese) subjects, supporting the theory under clinical dilemma that “the body weight may have an impact on rivaroxaban's PK profile, suggesting a need for dosing adjustment”.^24^, ^35^, ^36^, ^37^

Despite the ongoing research regarding DOACs, including rivaroxaban dosing in morbidly obese population, there is a lack of clinical consensus regarding this matter, which is largely driven by the fact that most of the reported studies on rivaroxaban PK in obese subjects suffer one or more of the following drawbacks: studies performed based on retrospective data,^24^, ^36^, ^38^, ^39^ based on single timepoint concentration measurement which does not allow for full PK profiling,^25^, ^35^, ^37^, ^39^ conducted using rivaroxaban sub‐therapeutic doses,^31^ based on a small number of obese subjects in the sample,^25^, ^32^, ^36^ missed to report distinctive results,^32^, ^33^, ^40^ had variable dose regimen of rivaroxaban,^25^, ^32^, ^33^, ^34^, ^40^ lacked a control or comparison group in the study design,^24^, ^25^, ^32^, ^33^, ^34^, ^35^, ^36^, ^37^, ^39^ or missed to have PK and the corresponding PD profiles for the same study sample.^25^, ^32^, ^34^, ^35^, ^36^, ^37^, ^39^ These limitations of the previous PK studies have collectively resulted in weak data to guide a conclusive or definite evidence about rivaroxaban's PK profile and dosing regimens in the obese population.^24^, ^28^, ^32^ For example, the International Society of Thrombosis and Hemostasis (ISTH) has issued a guidance statement concerning the use of DOACs among obese patients in 2016 as follows: “*We recommend appropriate standard dosing of the DOACs in patients with a BMI ≤40 kg/m* ^*2*^ *and weight ≤120 kg for VTE treatment, VTE prevention, and prevention of ischemic stroke and systemic arterial embolism in non‐valvular AF*”.^29^ Furthermore, the guidelines issued a guidance statement concerning the use of DOACs among morbidly obese patients as follows: “*We suggest that DOACs should not be used in patients with a BMI of >40 kg m* ^*2*^ *or a weight of >120 kg, because there are limited clinical data available for patients at the extreme of weight, and the available PK/PD evidence suggests that decreased drug exposures, reduced peak concentrations and shorter half‐lives occur with increasing weight, which raises concerns about underdosing in the population at the extreme of weight*”.^29^ Most recently in 2021, guideline recommendations on DOACs use in extreme obesity were updated,^27^ in which the use of the term “suggest” reflects a weak guidance statement because of limited existing literature. The statement reads: “*For treatment (or primary prevention) of VTE, we suggest that standard doses of rivaroxaban or apixaban are among appropriate anticoagulant options regardless of high BMI and weight*.” This highlights the ongoing uncertainty regarding rivaroxaban dosing in extreme obesity and the need for further investigation.

Therefore, the objectives of this study were to compare rivaroxaban PK and the coagulation profiles in obese healthy participants with body mass index (BMI) ≥35 kg/m^2^ (obese class II and III) vs. those of non‐obese healthy participants with BMI of 18.5 to <25 kg/m^2^. To the best of our knowledge, this is among the few prospective, interventional, and controlled clinical trials, aimed at investigating the PK and coagulation profiles of rivaroxaban in obese vs. non‐obese subjects using a therapeutic dose of rivaroxaban.

## METHODS

### Study design and setting

RIVOBESE‐PK is a non‐randomized, controlled, open‐label, parallel‐group, single‐dose, fed‐state clinical trial. Healthy volunteers who met the study's eligibility criteria were assigned into one of the two study groups based on their BMI: (1) obese group (healthy volunteers with BMI ≥35 kg/m^2^) or; (2) non‐obese group (healthy volunteers with BMI 18.5–24.9 kg/m^2^). The study was conducted at the International Center for Bioavailability, Pharmaceutical and Clinical Research (ICBR) in Cairo, Egypt. This trial was registered under the International Standard Randomized Controlled Trial Number (ISRCTN) registry (ISRCTN identifier: 12520248).

### Study population

Healthy male subjects with BMI 18.5–24.9 kg/m^2^ or >35 kg/m^2^ and aged between 18 and 60 years were enrolled into the study after passing the screening examinations which took place 3 weeks before the commencement of the study. Subjects were excluded if they had any: coagulation disorder, known increased bleeding risk, diagnosed chronic medical condition, or severe renal/hepatic impairment. A sample size of 36 in total (i.e., 18 subjects in each group) was calculated using independent‐sample *t*‐test to reliably (with probability greater than 0.8) detect an effect size, δ of 1.0, assuming a two‐sided criterion of detection that allows for a maximum Type I error rate, α of 0.05.^41^

Upon signing an informed consent form, each enrolled participant received a badge with a unique code which indicates his group (obese vs. non‐obese) and his sequence in that group. Participants were admitted to the clinical facility in ICBR at 8:00 PM, on the day prior to rivaroxaban dosing. All participants were checked for their body temperature, vital signs, and drug abuse. The recruited participants remained in the clinical facility under protocol conditions until the collection of the 18‐h post‐dose blood and urine samples. All food and fluid intake during the study was standardized for all participants 12 h prior to dosing, and up to 18 h post‐dosing. Adverse events and serious adverse drug reactions were assessed during the study for all participants.

### Study intervention and samples collection and processing

After an overnight fast, 11 h prior to drug intake, a single 20 mg film‐coated rivaroxaban tablet (Xarelto® Bayer, batch number: BXJLLK1, expiry date: 08/03/2024) was given orally with 240 mL water after a high‐fat and high‐calorie standardized breakfast to each participant in the two groups. Thirty minutes before rivaroxaban dose administration (i.e., at 7:30 AM), participants were required to eat a standardized breakfast meal. The subjects were instructed to eat their breakfast meal within 30 min or less. The maximum reported variability in rivaroxaban administration time between the participants was 12 min. Under direct supervision of the principal investigator and the clinical trial administrator, a total of 19 blood samples (78 mL) were withdrawn from each subject by certified nurses, pre‐dosing and then at 1, 2, 4, 8, 12, 18, 36, and 48 h post‐dosing. Blood samples were centrifuged at 3000 × *g* for 8 min, and the plasma was aliquoted and stored at −86°C until analysis. Actual blood sampling timepoints were recorded in each participant's case report form.

Furthermore, urine samples were obtained from each participant within the following time intervals: −2 to 0 h (pre‐dosing), and 0–3, 3–6, 6–9, 9–12, 12–15, 15–18 h post‐dosing. All urine samples collected during each time interval were pooled, and the total volume of the pooled urine in each interval was recorded. Out of the pooled urine, 10 mL of each interval was stored at −86°C until analysis. Actual urine collection timepoints and volumes were recorded in the urine samples collection form.

### Rivaroxaban analysis

Rivaroxaban extraction from plasma and urine samples was conducted by spiking the samples with a known concentration of rivaroxaban–d4 (internal standard). The procedure was carried out using acetonitrile for protein precipitation.^42^, ^43^, ^44^, ^45^, ^46^, ^47^, ^48^ Rivaroxaban concentrations in the processed samples were analyzed using a fully validated ultra‐performance liquid chromatography coupled with tandem mass spectrometer (UPLC‐MS/MS; ACQUITY H‐Class system, Waters, USA). The stationary phase consisted of C18 reversed phase column (Acquity UPLC BEH, 2.1 × 50 mm, 1.7 μm particle size) kept at 45°C. For plasma samples analysis, a mobile phase of 50% acetonitrile and 50% of a mixture of 10 mM ammonium acetate and 0.1% formic acid was delivered at a flow rate of 0.3 mL/min for a run time of 1.0 min. Similarly, for urine sample analysis, a mobile phase of 50% acetonitrile and 50% of 0.1% ammonia was delivered at a flow rate of 0.3 mL/min for a run time of 1.6 min.

The mass spectrometer was operated using an electrospray ionization source in positive mode. A 3.0 μL of the extracted samples was injected into the UPLC‐MS/MS and quantification was performed using transitions of m/z 436.14 → 144.96 for rivaroxaban, and 440.24 → 145.02 for rivaroxaban‐d4. The established range was linear over a rivaroxaban concentration range of 2.5–1000.0 and 10.0–30000.0 ng/mL, with a lower limit of quantification (LLOQ) of 2.5 and 10.0 ng/mL in plasma and urine, respectively. MassLynx version 4.1 software (Waters, Milford, MA, USA) was used for chromatography data acquisition and integration.

### Pharmacokinetic assessment

The PK parameters were calculated by employing Phoenix WinNonlin version 8.1 software using non‐compartmental model analysis, model 200 for plasma and 210 for urine (Certara, Princeton, NJ, USA). The primary PK parameters estimated were maximum plasma concentration (*C*_max_), time to reach *C*_max_ (*t*_max_), area under the plasma concentration vs. time curve from zero to 48 h (AUC_0–48_) and from zero to infinity (AUC_0–inf_), elimination rate constant (*k*_*e*_), half‐life (*t*_1/2_), mean residence time from zero to 48 h (MRT_0–48_) and from zero to infinity (MRT_0–inf_), apparent volume of distribution (*V*_*d*_/*F*), apparent clearance (Cl/*F*), and fraction of dose recovered unchanged in urine over the urine collection period (*f*_*e*_).

Using the linear regression curve of log concentration versus time of at least three timepoints in the terminal phase of the curve, *k*_*e*_ was obtained, and accordingly, *t*_1/2_ was calculated as 0.693/*k*_*e*_, assuming a linear terminal phase of the PK profiles. The AUC was calculated from the concentration vs. time curve using the linear trapezoidal rule.^49^ By extrapolating the curve to infinity, the AUC_0–inf_ was calculated using Equation (1) below. Apparent total body clearance (Cl/*F*) and volume of distribution (*V*_*d*_/*F*) were calculated using Equations (2) and (3), respectively.

(1) $${\mathrm{AUC}}_{0–\inf}={\mathrm{AUC}}_{0–48}+\left(C^{48}/k_{e}\right)$$

(2) $$\mathrm{Cl}/\text{F}=\text{Dose}/{\mathrm{AUC}}_{0–\inf}$$

(3) $$V_{d}/F=\left(\mathrm{Cl}/F\right)/k_{e}=\text{Dose}/\left({\mathrm{AUC}}_{0–\inf}\times k_{e}\right)$$

Rivaroxaban amount excreted in urine during each of the collection periods over 18 h post‐dose administration was estimated based on the measured rivaroxaban concentration in urine and the voided urine volume; and this was expressed as a fraction of the administered dose (*f*_*e*_). The obtained PK parameters were evaluated at two different levels for triangulation purposes. First, PK parameters were statistically compared between the obese group (BMI ≥35 kg/m^2^; *n* = 18) and non‐obese group (BMI 18.5–24.9 kg/m^2^; *n* = 18). Second, PK parameters were statistically compared between the morbidly obese group (with BMI >40 kg/m^2^ and/or body weight >120 kg; *n* = 6) and non‐obese group (BMI 18.5–24.9 kg/m^2^; *n* = 18).

### Coagulation profile assessment

The coagulation profile assessment including prothrombin time (PT), the corresponding international normalized ratio (INR), and activated partial thromboplastin clotting time (aPTT) were obtained for all participants. Two mL of blood was withdrawn pre‐dosing and at 1, 2, 4, 8, 12, 18, 36, and 48 h post‐dosing in sodium citrate tubes (total 18 mL) to perform these coagulation assays (PT and aPTT).

### Statistical analysis

Statistical analyses were performed utilizing Statistical Package for the Social Sciences (SPSS, version 28). The independent samples *t*‐test was used to compare *C*_max_, AUC_0–48_, AUC_0–inf_, *t*_1/2_, Cl/*F*, and *f*_*e*_ in obese vs. non‐obese subjects. Mann–Whitney U test was used to compare *t*_max_, *k*_*e*_, MRT_0–48_, MRT_0–inf_, *V*_*d*_/*F* between the two groups. Simple linear and multiple linear regressions were used to detect any potential associations between the variables (to account for any confounding factors). The level of significance was set at 0.05 for all statistical analyses, except for simple linear regression, which was set at 0.2.

### Ethical approvals

The study was conducted in accordance with ICH‐GCP and the Declaration of Helsinki ethical principles. The study protocol was approved by the independent ethics committee of the ICBR (FORM04/SOP: QA‐034 – RESH‐012). Moreover, ethical approvals were obtained from Egypt Drug Authority, the Evaluation Unit of Bioavailability and Bioequivalence Studies for Human Pharmaceuticals, and from the Qatar University Institutional Review Board (approval number: QU‐IRB 1741‐E/22).

## RESULTS

**FIGURE 1:**
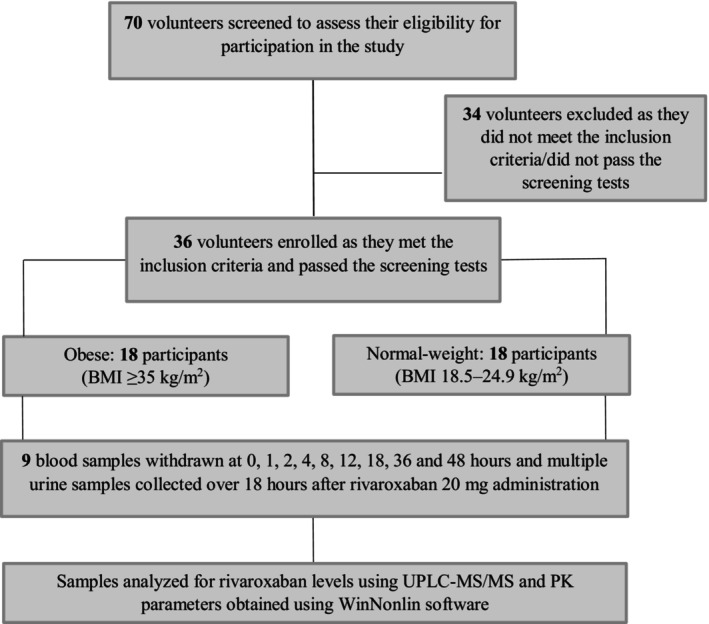
Flowchart diagram of the study workflow.

Seventy volunteers were screened, of whom 36 met the eligibility criteria and assigned to one of the two groups based on BMI: obese group (BMI ≥35 kg/m^2^) and non‐obese group (BMI 18.5–24.9 kg/m^2^); all participants have completed the study per protocol, with no dropouts (Figure 1). Recruitment of study participants has taken place in the period between July 1st to July 31st 2022.

Baseline demographic characteristics and laboratory examinations were similar between the groups, except for age, alanine aminotransferase (ALT), aspartate aminotransferase (AST), and fasting plasma glucose level (*p* = 0.007, 0.019, 0.034, and 0.005, respectively), which were significantly higher in the obese participants (Table 1). However, neither simple linear regression analysis nor multiple linear regression analysis has shown an association between these variables and the *C*_max_, eliminating the probability of these variables' effect on the *C*_max_. Rivaroxaban was well‐tolerated and no deaths or adverse events were reported in the study participants of both groups.

**TABLE 1:** Participants' demographic characteristics and laboratory examinations at baseline (*n* = 36).

Characteristics^d^	Total (*n* = 36)	Obese (*n* = 18)	Non‐obese (*n* = 18)	*p*‐value
Age (years)	31.1 (11.43)	35.6 (11.7)	26.6 (9.5)	0.007^a^
Body weight (kg)	89.5 (24.3)	111.5 (11.7)	67.4 (7.3)	<0.001^a^
BMI (kg/m^2^)	29.6 (7.9)	37.0 (2.6)	22.2 (2.2)	<0.001^a^
Smokers, *n* (%)	17 (47.2)	6 (33.33)	11 (61.11)	0.095^b^
Total bilirubin (mg/dL)	0.733 (0.29)	0.731 (0.301)	0.736 (0.28)	0.995^c^
Direct bilirubin (mg/dL)	0.241 (0.088)	0.217 (0.061)	0.264 (0.105)	0.114^c^
Creatinine (mg/dL)	0.857 (0.104)	0.837 (0.096)	0.877 (0.111)	0.255^c^
Blood urea nitrogen (mg/dL)	27.92 (6.9)	27.1 (5.45)	28.7 (8.2)	0.491^c^
ALT (U/L)	24.28 (13.63)	30.33 (15.75)	18.2 (7.55)	0.019^a^
AST (U/L)	19.70 (7.6)	22.2 (8.04)	17.17 (6.37)	0.034^a^
Albumin (g/dL)	4.67 (0.2)	4.64 (0.17)	4.7 (0.22)	0.254^a^
Total cholesterol (mg/dL)	162.6 (36.2)	170.3 (38.98)	155.0 (32.5)	0.21^c^
Triglycerides (mg/dL)	104.78 (67.9)	123.7 (85.6)	85.9 (37.5)	0.097^a^
HDL (mg/dL)	43.2 (7.9)	41.8 (8.64)	44.67 (7.08)	0.280^c^
LDL (mg/dL)	98.5 (31.6)	103.78 (35.4)	93.2 (27.2)	0.320^c^
Fasting plasma glucose (mg/dL)	88.31 (8.3)	92.1 (7.97)	84.6 (6.96)	0.005^c^

Abbreviations: ALT, alanine aminotransferase; AST, aspartate aminotransferase; BMI, body mass index; HDL, high‐density lipoprotein; LDL, low‐density lipoprotein.

^a^ Mann–Whitney U test.

^b^ Chi‐square test.

^c^ Independent sample *t*‐test.

^d^ Values presented as mean (SD) unless otherwise indicated.

**FIGURE 2:**
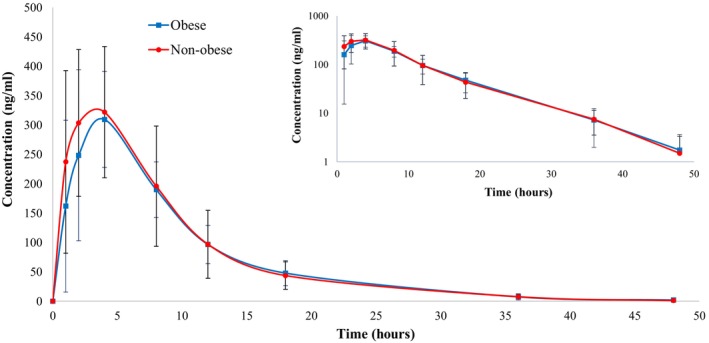
Mean plasma concentrations of rivaroxaban versus time curves following a dose of rivaroxaban 20 mg [obese with BMI ≥35 kg/m^2^ (*n* = 18) vs. non‐obese with BMI 18.5–24.9 kg/m^2^ (*n* = 18)]. The concentrations shown on the graph are the averages of the individuals' concentrations at each sampling timepoint. Inlet figure presents data on log‐scale y‐axis and shows a linear elimination terminal phase. This figure is Excel Microsoft‐generated.

The mean plasma concentration vs. time profiles of rivaroxaban after a single oral dose of 20 mg is shown in Figure 2 and the PK parameters are summarized in Table 2. Insignificant decrease of 13.5% in the *C*_max_ was observed in obese compared with non‐obese participants (339.7 ± 84.2 vs. 392.9 ± 78.9 ng/mL; *p* = 0.059). Moreover, no significant differences between the two groups were found in other PK parameters, including *t*_max_, AUC_0–48_, AUC_0–inf_, *k*_*e*_, *t*_1/2_, MRT_0–48_, MRT_0–inf_, *V*_*d*_/*F*, and Cl/*F*.

**TABLE 2:** Pharmacokinetic parameters estimated from plasma data in obese (with BMI >35 kg/m^2^) and non‐obese (with BMI 18.5–24.9 kg/m^2^) subjects at baseline and up to 48 h following an oral administration of rivaroxaban 20 mg (*n* = 36).

PK parameter^c^	Obese (*n* = 18)	Non‐obese (*n* = 18)	*p*‐value
*C*_max_ (ng/mL)	339.7 (84.2)	392.9 (78.9)	0.059^a^
*t*_max_ (h), median (25th–75th percentile)	4.00 (2–4)	2.00 (2–4)	0.303^b^
AUC_0–48_ (ng∙h/mL)	3339.2 (872.9)	3534.5 (805.3)	0.490^a^
AUC_0–inf_ (ng∙h/mL)	3481.1 (809.2)	3595.3 (805.7)	0.678^a^
*k*_*e*_ (1/h), median (25th–75th percentile)	0.10 (0.09–0.13)	0.11 (0.08–0.12)	0.569^b^
*t*_1/2_ (h)	6.61 (1.4)	7.2 (1.77)	0.310^a^
MRT_0–48_ (h), median (25th–75th percentile)	8.22 (7.55–9.82)	7.46 (6.97–8.82)	0.088^b^
MRT_0–inf_ (h), median (25th–75th percentile)	9.19 (8.12–10.64)	8.14 (7.64–9.91)	0.176^b^
*V*_*d*_/*F* (L), median (25th–75th percentile)	60.38 (45.9–63.5)	56.88 (45.3–72.3)	0.817^b^
Cl/*F* (L/h)	6.04 (1.38)	5.812 (1.22)	0.614^a^

Abbreviations: AUC_0–48_, area under concentration–time curve from time zero to the last measurable concentration at 48 h; AUC_0–inf_, area under concentration–time curve from time zero to infinity; Cl/*F*, apparent clearance; *C*_max_, mean ± SD of maximum plasma concentration of each participant; *k*_*e*_, elimination rate constant; MRT, mean residence time; *t*_1/2_, half‐life; *t*_max_, mean ± SD of the time at which maximum plasma rivaroxaban concentration was achieved for each participant; *V*_*d*_/*F*, apparent volume of distribution.

^a^ Independent sample *t*‐test.

^b^ Mann‐Whitney U test.

^c^ Values presented as mean (standard deviation) unless otherwise indicated.

**FIGURE 3:**
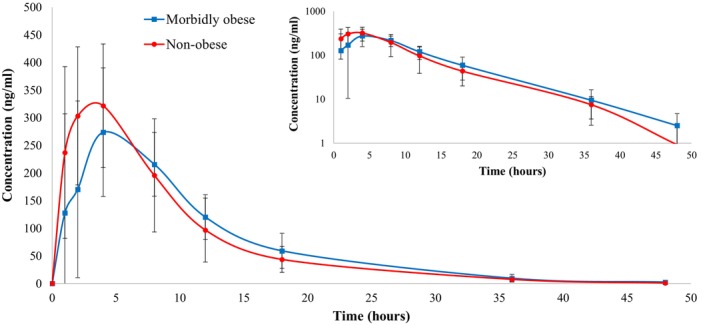
Mean plasma concentrations of rivaroxaban versus time curve following a dose of rivaroxaban 20 mg [morbidly obese with BMI >40 kg/m^2^ and/or body weight >120 kg (*n* = 6) vs. non‐obese with BMI 18.5–24.9 kg/m^2^ (*n* = 18)]. The concentrations shown on the graph are the averages of the individuals' concentrations at each sampling timepoint. Inlet figure presents data on log‐scale y‐axis and shows a linear elimination terminal phase. This figure is Excel Microsoft‐generated.

As recommended by ISTH and with the intention of investigating the PK parameters of rivaroxaban in morbidly obese participants solely, a subgroup analysis including all morbidly obese participants (BMI >40 kg/m^2^ and/or body weight >120 kg) was performed. These morbidly obese participants (*n* = 6) were compared with the control group which included all participants with normal body weight or BMI (*n* = 18) (BMI 18.5–24.9 kg/m^2^). The subgroup analysis was performed using the same prespecified statistical analysis tests, as described in the Methods section. The *C*_max_, *t*_max_, AUC_0–48_, AUC_0–inf_, *k*_*e*_, *t*_1/2_, MRT_0–48_, MRT_0–inf_, *V*_*d*_/*F*, and Cl/*F* values revealed no statistically significant difference between the two groups (refer to Table 3 and Figure 3).

**TABLE 3:** Pharmacokinetic parameters estimated from plasma data in morbidly obese (with BMI >40 kg/m^2^ and/or body weight >120 kg) and non‐obese (with BMI 18.5–24.9 kg/m^2^) subjects at baseline and up to 48 h following an oral administration of rivaroxaban 20 mg (*n* = 24).

PK parameter^c^	Morbidly obese (*n* = 6)	Non‐obese (*n* = 18)	*p*‐value
*C*_max_ (ng/mL)	317.3 (101.6)	392.9 (78.9)	0.071^a^
*t*_max_ (h), median (25th –75th percentile)	4.00 (1.8–8)	2.00 (2–4)	0.310^b^
AUC_0–48_ (ng∙h/mL)	3500.7 (1271.2)	3534.5 (805.3)	0.939^a^
AUC_0–inf_ (ng∙h/mL)	3834.8 (1174.9)	3595.3 (805.7)	0.599^a^
*k*_*e*_ (1/h), median (25th–75th percentile)	0.10 (0.09–0.27)	0.11 (0.08–0.12)	0.721^b^
*t*_1/2_ (h)	6.7 (0.9)	7.2 (1.77)	0.614^a^
MRT_0–48_ (h), median (25th–75th percentile)	10.49 (8.0–11.77)	7.46 (6.97–8.82)	0.137^b^
MRT_0–inf_ (h), median (25th–75th percentile)	12.15 (9.0–12.4)	8.14 (7.64–9.91)	0.055^b^
*V*_*d*_/*F* (L), median (25th–75th percentile)	46.68 (38.9–76.4)	56.88 (45.3–72.3)	0.587^b^
Cl/*F* (L/h)	5.65 (1.8)	5.812 (1.22)	0.817^a^

Abbreviations: AUC_0–48_, area under concentration–time curve from time zero to the last measurable concentration at 48 h; AUC_0–inf_, area under concentration–time curve from time zero to infinity; Cl/*F*, apparent clearance; *C*_max_, mean ± SD of maximum plasma concentration of each participant; *k*_*e*_, elimination rate constant; MRT, mean residence time; *t*_1/2_, half‐life; *t*_max_, mean ± SD of the time at which maximum plasma rivaroxaban concentration was achieved for each participant; *V*_*d*_/*F*, apparent volume of distribution.

^a^ Independent sample *t*‐test.

^b^ Mann‐Whitney U test.

^c^ Values presented as mean (standard deviation) unless otherwise indicated.

For urine data, no statistically significant difference was found between the two groups in terms of the mean *f*_*e*_ over the collection period (0.289 ± 0.088 vs. 0.242 ± 0.082). Table 4 represents a detailed description of the urine data for both groups.

**TABLE 4:** Urine data were obtained in obese (with BMI >35 kg/m^2^) and non‐obese (with BMI 18.5–24.9 kg/m^2^) subjects during the collection period (18 h) following an oral administration of rivaroxaban 20 mg (*n* = 36).

Interval No.	Interval time		Time period (h)	Mid‐point time (h)	Mass recovered (mg), mean (SD)	Average excretion rate (mg/h), mean (SD)	*f*_*e*_ over the collection period (18 h), mean (SD)
	Beginning	Ending					
Obese (*n* = 18)							
1	0.00	3.32	3.32	1.66	1.32 (0.87)	0.40 (0.26)	0.289 (0.088)
2	3.32	6.11	2.79	4.72	1.50 (0.57)	0.54 (0.21)	
3	6.11	9.22	3.11	7.67	1.36 (0.63)	0.44 (0.21)	
4	9.22	12.21	2.99	10.72	0.87 (0.32)	0.29 (0.10)	
5	12.21	15.24	3.03	13.73	0.49 (0.23)	0.16 (0.79)	
6	15.24	18.28	3.05	16.76	0.25 (0.13)	0.07 (0.04)	
Non‐obese (*n* = 18)							
1	0.00	3.30	3.38	1.692	1.41 (0.74)	0.42 (0.22)	0.242 (0.082)
2	3.38	6.10	2.72	4.74	1.03 (0.47)	0.38 (0.17)	
3	6.10	9.21	3.09	7.65	1.14 (0.71)	0.37 (0.24)	
4	9.22	12.23	2.01	10.73	0.71 (0.47)	0.24 (0.15)	
5	12.20	15.24	3.02	13.71	0.36 (0.17)	0.12 (0.06)	
6	15.22	18.21	2.99	15.88	0.20 (0.117)	0.07 (0.039)	

*Note*: *f*_*e*_: fraction of dose recovered unchanged in urine over 18 h post‐dosing.

**FIGURE 4:**
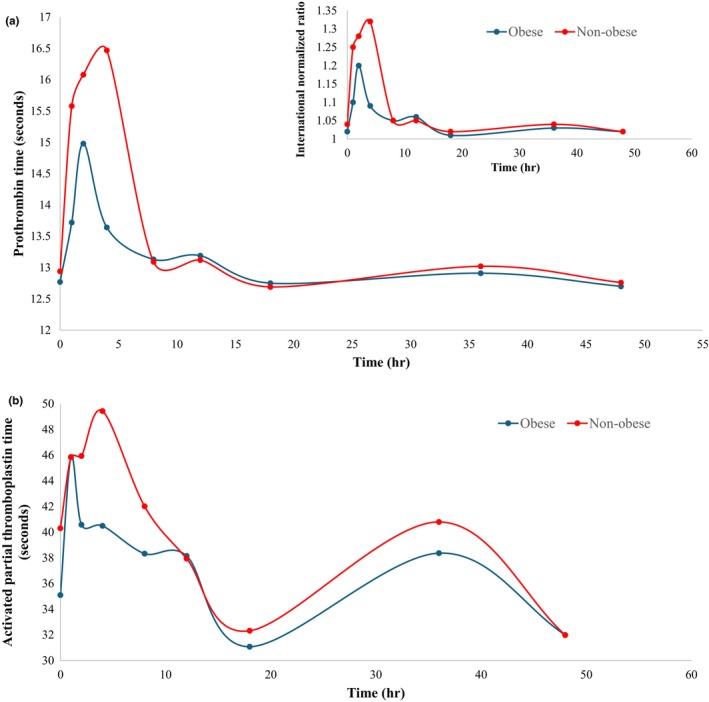
(a) Prothrombin time (and the international normalized ratio, inlet figure) at baseline and up to 48 h following an oral administration of rivaroxaban 20 mg (*n* = 36); (b) Activated partial thromboplastin time at baseline and up to 48 h following an oral administration of rivaroxaban 20 mg (*n* = 36).

Statistical analysis of the coagulation profile revealed a significant difference between obese and non‐obese groups with respect to PT measurements at 1 h (13.72 vs. 15.58, *p* = 0.013) and 4 h (13.64 vs. 16.47, *p* = 0.008) (Figure 4). Similarly, the corresponding INR measurements have shown a significant difference between the two groups at 1 h (1.10 vs. 1.25, *p* = 0.013), and 4 h (1.09 vs. 1.32, *p* = 0.008). In contrast, no significant difference was found for the aPTT at any timepoint between the two groups.

## DISCUSSION

Across the literature, a limited number of studies have traditionally investigated rivaroxaban PK profile in the obese population.^24^, ^25^, ^31^, ^33^, ^34^, ^35^, ^36^ The evidence generated by these studies is inconclusive, and consequently, the controversy about rivaroxaban dosing in the obese population persists. Hence, more robust prospective controlled studies, which investigate rivaroxaban pharmacokinetics in obese subjects are highly recommended by the recent guidelines.^27^ In light of this, the current clinical PK trial was conducted aiming to investigate the PK and the coagulation profiles of rivaroxaban in obese participants with BMI ≥35 kg/m^2^ when compared with normal‐weight participants with BMI of 18.5 to <25 kg/m^2^. In summary, two groups of participants were enrolled in the study and received a single dose of 20 mg rivaroxaban film‐coated tablet as the study intervention. Rivaroxaban concentrations were measured in plasma and urine samples, and the PK parameters (i.e., *C*_max_, *t*_max_, AUC_0–48_, AUC_0–inf_, *k*_*e*_, *t*_1/2_, MRT_0–48_, MRT_0–inf_, *V*_*d*_/*F*, Cl/*F*, and *f*_*e*_) and coagulation profiles (i.e., PT, INR, and aPTT) were determined and compared between the two groups. The main analysis (*n* = 36) revealed no significant difference between the two groups in terms of *C*_max_, *t*_max_, AUC_0–48_, AUC_0–inf_, *k*_*e*_, *t*_1/2_, MRT_0–48_, MRT_0–inf_, *V*_*d*_/*F*, Cl/F, and *f*_*e*_. Moreover, subgroup analysis for participants with BMI >40 kg/m^2^ and/or weight >120 kg (*n* = 6) compared to those with BMI 18.5 to <25 kg/m^2^ (*n* = 18) yielded similar results.

The results showed that *C*_max_ was comparable in obese and non‐obese participants (*p* = 0.059). In obese participants, the *C*_max_ was 339.7 ng/mL, which was well substantiated by the *C*_max_ value (305.0 ng/mL) following 20 mg rivaroxaban reported by Speed et al. in obese subjects with an average weight of 125 kg.^25^ Moreover, the *C*_max_ range for obese participants (207–485 ng/mL) in the current study was comparable to the *C*_max_ range (200–350 ng/mL) reported in a previous study.^36^ On the other hand, one previous study has reported lower *C*_max_ of 214 and 220 ng/mL for AF and VTE cohorts with a median body weight of 139 kg and a BMI ≥40 kg/m^2^ respectively.^35^ This difference in *C*_max_ may be attributed to the difference in body weight and BMI (i.e., 111.5 kg, 37.0 kg/m^2^ in the current study), a different technique used for rivaroxaban concentration measurement (chromogenic assays vs. UPLC), and to the fact that *C*_max_ was determined using one sampling timepoint within 2–4 h post‐rivaroxaban dosing in that previous study.

To be able to compare to the literature, the AUC_0–24_ median [range] value of obese participants in our study was calculated to be 3214.5 [2009–4749] ng.hr/mL, the value which was almost double the AUC_0–24_ reported by a previous study (1204 [861–1390] ng∙h/mL).^24^ Differences in AUC_0–24_ median values between the current study and the indicated previous study might be due to the differences in the study participants (exclusively male healthy with a mean BMI of 37 kg/m^2^ in this study vs. 60% male AF patients with a mean BMI of 44 kg/m^2^), and the different rivaroxaban level determination techniques (UPLC vs. anti‐factor Xa assay, respectively).^24^ Another previous study that retrospectively determined AUC_0–24_ via PK simulation from single timepoints in patients, reported the mean of AUC_0–24_ to be 2800 ng∙h/mL,^25^ which is lower than the obtained value in the current study (3144.2 ng∙h/mL). The observed variations in AUC_0–24_ mean values between this study and the indicated previous study could be attributed to the study design (prospective vs. retrospective, multiple timepoints vs. single timepoint, and observed vs. predicted PK profiles). Similarly, for AUC_0–inf_, a higher value of 3481.1 ng∙h/mL was reported in the present study compared with 1155 ng∙h/mL reported in a prior study on subjects >120 kg.^50^ This difference could be attributed to the different doses investigated in each study (20 mg vs. 10 mg), and different population ancestry (Egyptians vs. Caucasian). A median *V*_*d*_/*F* of 60.38 L in obese participants in this study was comparable to previously reported values of 73.40 and 82.80 L.^24^, ^31^ To be able to compare our apparent clearance value to the literature, the median value in obese participants was calculated (5.63 L/h), the value which was relatively lower than a previously reported median value of 16.80 L/h,^24^ in which blood samples were obtained during steady‐state which might be a possible reason for the difference in the clearance values.^24^ On the other hand, the mean apparent clearance for obese subjects reported by a previous study^31^ was 7.86 L/h, which is comparable to 6.04 L/h in this study. It is noteworthy that the results obtained from the subgroup analysis (*n* = 24) align with those of the main analysis (*n* = 36), which emphasizes that the conclusion of “no difference in PK parameters of rivaroxaban” applies to the morbidly obese subjects, comprising 33% of the study sample. Regarding the urine data, extrapolating the *f*_*e*_ values obtained in this study from the 18‐h urine collection period to 48‐h showed that *f*_e_ can reach up to 43% and 35% for obese and non‐obese populations, respectively, which are in line with the 36% reported *f*_e_ for the rivaroxaban dose excreted unchanged in urine for the general population.^20^ To corroborate these points, the comparison of the PK parameters reported in our study with those reported previously was challenging due to substantial variations in study design, participants' characteristics, rivaroxaban dose, sampling timepoints, and rivaroxaban concentration measurement techniques.

Interestingly, despite the known differences between obese and non‐obese subjects in terms of body fat composition and the consequent expected changes in the distribution and resulting PK profile and parameters of lipophilic drugs, the findings revealed no differences (obese or morbidly obese vs. non‐obese). However, the results demonstrated a decreasing trend (although not significant) of *C*_max_ in the obese (339.7 ng/mL) and morbidly obese (317.3 ng/mL) compared with the non‐obese (392.9 ng/mL) populations. Also, a longer *t*_max_ values (although not significant) were observed in the obese (4 h) and morbidly obese (4 h) populations compared with the non‐obese (2 h) population. In addition, an increasing trend (although not significant) of MRT_0–inf_ in the obese (9.19 h) and morbidly obese (12.15 h) compared with the non‐obese (8.14 h) populations was observed. This could be attributed in part to rivaroxaban being in BCS II (low solubility and high permeability) with moderate lipophilicity (log *p* = 1.5)^20^ reflecting low‐to‐medium affinity to peripheral tissues, which might lead to distribution in the excess fat tissues within morbidly obese, although not supported by the current reported *V*_d_ data (56.88, 60.38, 46.68 L in the non‐obese, obese, and morbidly obese populations, respectively) or the AUC_0–inf_ data (3595.3, 3481.1, and 3834.8 ng.hr/mL in the non‐obese, obese, and morbidly obese populations, respectively).

Regarding the coagulation profile of rivaroxaban, PT, INR, and aPTT coagulation tests were performed at baseline and up to 48 h post‐dosing. Comparisons with reported studies were not feasible due to the absence of any previous studies focusing on assessing the coagulation profile of rivaroxaban in the obese population. While PT, INR, and aPTT are known to be influenced by rivaroxaban levels, their sensitivity for assessing rivaroxaban's PD activity are limited compared with dedicated assays (i.e., anti‐Xa assay). Therefore, the results of these coagulation tests in this study may not accurately reflect the true extent of rivaroxaban's anticoagulant effect.^19^, ^51^, ^52^ Although PT and aPTT have not been demonstrated to measure the anticoagulation activity of rivaroxaban, it is worth mentioning that these coagulation tests are prolonged by rivaroxaban in a dose‐dependent manner and thus would inform clinical decisions only in emergency cases, that is, bleeding or urgent invasive therapy need.^19^, ^51^ All coagulation profiles investigated in this study were used to compare between the obese and non‐obese populations. However, the use of these tests in clinical practice settings for coagulation activity monitoring can lead to false interpretations.^52^

The efficacy and safety of rivaroxaban in the obese population have been investigated across the literature. For example, a post hoc analysis, stratified by BMI, was conducted in a cohort receiving rivaroxaban for the prevention of stroke and embolism in AF (ROCKET AF^53^), and it revealed that the risk of stroke in the obese group (BMI ≥35 kg/m^2^) is significantly lower than that in the normal‐weight group while the bleeding level was similar in both groups.^54^ Similarly, a sub‐study of EINSTEIN DVT and EINSTEIN PE trials of rivaroxaban^55^, ^56^, ^57^ indicated no association between BMI and risk of recurrent VTE or major bleeding.^58^ More recently, one cohort study investigated the efficacy and safety of rivaroxaban and apixaban in non‐valvular AF and VTE patients and revealed no significant difference across all BMI groups regarding stroke incidences, VTE recurrence rates, and major and non‐major bleeding.^59^

It is plausible that a few limitations in this study could have influenced our results. First, recruitment of female volunteers was not possible due to the hospitalization for over 18 h including an overnight stay, which was inconvenient to females in our setting and thus, only male volunteers were recruited. Secondly, this study has included obese class II and III patients, but with a majority of a BMI <40 kg/m^2^, and therefore, there is a likelihood that findings may have been skewed. Should obese class III only be recruited, significant differences in PK parameters are possible. Thirdly, urine samples were collected from participants for up to 18 h (around 3 × *t*_1/2_ only); therefore, partial capture of renal excretion was achieved and further urine samples for up to at least 5 × *t*_1/2_ would be needed to determine accurately 95–99% of rivaroxaban excreted unchanged in urine. Fourthly, while the study exhibits a controlled, parallel‐group design, it is worth noting that a single dose might, to some extent, not fully reflect the PK parameters that could be observed in a more comprehensive, long‐term (multiple‐dose) design. All aforementioned limitations would affect the generalizability of the study.

In summary, in this prospective controlled clinical trial, PK profiles after oral rivaroxaban 20 mg were mostly similar in obese compared with non‐obese participants. Maximum rivaroxaban concentration and exposure inferred by AUC were not affected in the obese participants after single dosing in the period of the study, that is, 48 h. However, multiple dosing with a longer study period could lead to different conclusions. Taken together, this study provides valuable insights regarding the use of rivaroxaban in the obese population. While our findings contribute to the current available evidence, it is important to acknowledge the limitations of our study and the complexities of clinical practice. Further research is warranted to further demonstrate the optimal management strategies for this patient population.

## AUTHOR CONTRIBUTIONS

All authors wrote the manuscript; M.A., A.A., O.R., and I.A. designed the research; M.A., A.A., O.R., H.E., M.D., K.M., and F.I. performed the research; M.A., A.A., O.R., A.E., and Y.E analyzed the data.

## FUNDING INFORMATION

The study was funded by [Qatar University]: Student Grants (QUST‐1‐CPH‐2023‐843, QUST‐2‐ CPH ‐2021‐169, QUST‐2‐CPH‐2020‐13) and QU Health Pump Priming Grant.

## CONFLICT OF INTEREST STATEMENT

The authors declared no competing interests for this work.
